# Mouse Bone Marrow-Derived Mesenchymal Stromal Cells Turn Activated Macrophages into a Regulatory-Like Profile

**DOI:** 10.1371/journal.pone.0009252

**Published:** 2010-02-16

**Authors:** Julian Maggini, Gerardo Mirkin, Ianina Bognanni, Josefina Holmberg, Isabel M. Piazzón, Irene Nepomnaschy, Héctor Costa, Cristian Cañones, Silvina Raiden, Mónica Vermeulen, Jorge R. Geffner

**Affiliations:** 1 Instituto de Investigaciones Hematológicas, Academia Nacional de Medicina, Buenos Aires, Argentina; 2 Departamento de Microbiología, Parasitología e Inmunología, Facultad de Medicina, Universidad de Buenos Aires, Buenos Aires, Argentina; 3 Centro Nacional de Referencia para el SIDA, Facultad de Medicina, Universidad de Buenos Aires, Argentina; Institut de Pharmacologie et de Biologie Structurale, France

## Abstract

In recent years it has become clear that the therapeutic properties of bone marrow-derived mesenchymal stromal cells (MSC) are related not only to their ability to differentiate into different lineages but also to their capacity to suppress the immune response. We here studied the influence of MSC on macrophage function. Using mouse thioglycolate-elicited peritoneal macrophages (M) stimulated with LPS, we found that MSC markedly suppressed the production of the inflammatory cytokines TNF-α, IL-6, IL-12p70 and interferon-γ while increased the production of IL-10 and IL-12p40. Similar results were observed using supernatants from MSC suggesting that factor(s) constitutively released by MSC are involved. Supporting a role for PGE_2_ we observed that acetylsalicylic acid impaired the ability of MSC to inhibit the production of inflammatory cytokines and to stimulate the production of IL-10 by LPS-stimulated M. Moreover, we found that MSC constitutively produce PGE2 at levels able to inhibit the production of TNF-α and IL-6 by activated M. MSC also inhibited the up-regulation of CD86 and MHC class II in LPS-stimulated M impairing their ability to activate antigen-specific T CD4+ cells. On the other hand, they stimulated the uptake of apoptotic thymocytes by M. Of note, MSC turned M into cells highly susceptible to infection with the parasite *Trypanosoma cruzi* increasing more than 5-fold the rate of M infection. Using a model of inflammation triggered by s.c. implantation of glass cylinders, we found that MSC stimulated the recruitment of macrophages which showed a low expression of CD86 and the MHC class II molecule Ia^b^ and a high ability to produce IL-10 and IL-12p40, but not IL-12 p70. In summary, our results suggest that MSC switch M into a regulatory profile characterized by a low ability to produce inflammatory cytokines, a high ability to phagocyte apoptotic cells, and a marked increase in their susceptibility to infection by intracellular pathogens.

## Introduction

Bone marrow-derived mesenchymal stromal cells (MSC) are pluripotent adult stromal cells able to differentiate into different cell types such as osteoblasts, chondrocytes and adipocytes [1]. These cells preferentially home to damaged tissues and therefore they have attracted scientific attention as potential therapeutic tools for tissue repair [1]–[3]. Studies performed in the last years, however, demonstrated that MSC exert potent immunomodulatory effects and support the notion that the therapeutic potential of MSC is not only related to their ability to differentiate into different lineages but also to their capacity to suppress the immune response [4], [5].

A larger number of studies mainly performed in vitro have shown that MSC display immunomodulatory capacities. MSC inhibit the differentiation of monocytes and CD34+ haematopoietic progenitor cells into dendritic cells (DC) [6], [7]. They suppress the maturation of DC and the ability of DC to produce inflammatory cytokines as well as T helper-type 1 (Th1)-promoting cytokines such as IL-12p70 [6]–[9]. MSC inhibit cytotoxicity and production of interferon-γ by NK cells [10], [11], and also exert a potent immunosuppressive effect on T cells. They suppress T-cell proliferation induced by alloantigens, mitogens and soluble antigens [12]–[14]. Interestingly, this inhibition appears to be not MHC restricted as it can be induced by either autologous or allogeneic MSC. Other immunosuppressive effects mediated by MSC on T cells include down regulation of T-CD8+-mediated cytotoxicity [15] and expansion of regulatory T cells [16]. MSC are also able to regulate B cell function, suppressing both, proliferation of B cells in response to anti-Ig antibodies, soluble CD40, and cytokines, and antibody production [17], [18]. The mechanisms underlying the immunosuppressive effects mediated by MSC are not fully defined, but they appear to be largely mediated by a number of soluble factors produced by MSC, either constitutively or in response to paracrine signals derived from leukocytes. These soluble mediators include transforming growth factor-β1 (TGFβ1), hepatocyte growth factor, PGE_2_, indoleamine 2,3-dioxygenase (IDO), haem oxygenase-1, soluble HLA-G5, IL-10 and IL-6 [4], [5], [14].

Little is known about the effect of MSC on macrophages, a critical player of the innate immune response involved in almost all immune-mediated diseases. In the present study we analyzed this subject. We show here that MSC turns activated macrophages into a regulatory profile characterized by a low ability to produce inflammatory cytokines, a high ability to phagocyte apoptotic cells, and a dramatic increase in their susceptibility to infection with the parasite *Trypanosoma cruzi* (*T. cruzi*).

## Materials and Methods

### Mice

All experiments were conducted using 2-mo-old virgin female C57BL/6 mice or ovalbumin (OVA)-specific, MHC II-restricted, T-cell-receptor transgenic mice (OT-II mice). OT-II mice carry a transgenic CD4 TCR specific for the MHC class II-restricted OVA peptide aa 323–339. Mice were housed six per cage and kept at 20±2°C under automatic 12-h light-dark schedule. All animal experiments were performed according to the NIH Guide for the Care and Use of Laboratory Animals and approved by the ethical committee of the “Instituto de Leucemia Experimental” (ILEX) (Academia Nacional de Medicina, Buenos Aires, Argentina).

### Mouse MSC cultures

MSC were isolated and cultured using standard protocols [19]. Bone marrow cells from C57BL/6 mice were collected by flushing the femurs and tibias from 8–12-week-old mice with RPMI medium supplemented with 5% heat-inactivated fetal calf serum (FCS) (Invitrogen, CA, USA). Erythrocytes-depleted bone marrow cells were plated at a density of 4×10^6^ cells per cm^2^ in RPMI medium supplemented with 10% FCS, 100 IU/ml penicillin and 100 µg/ml streptomycin (Invitrogen). Culture medium was changed at day 2 to remove nonadherent cells. Whole medium was subsequently replaced weekly. The cells were grown for 3–4 weeks until almost confluent. Adherent cells were then detached by 0.25% trypsin-EDTA and replated using a 1∶3 dilution until passage 2. Subsequent passaging and seeding of the cells were performed at a density of 5,000 cells per cm^2^. MSC were used after 4–7 passages.

### Analysis of MSC

Osteogenic and adipogenic differentiation assays were performed as previously described [20]. For standard osteogenic differentiation, confluent monolayers of MSC were incubated in medium supplemented with 10^−8^ M dexamethasone, 50 µM ascorbic acid, and 10 mM β-glycerol phosphate (Sigma-Aldrich, Buenos Aires, Argentina) with changes of medium every 5 days. After 30 days the cultures were fixed with 70% cold ethanol for 1 h at room temperature, and incubated with Alizarin Red S (2% aqueous solution, pH 4.1–4.3, adjusted with ammonium hydroxide) for 30 min. Excess stain was removed by washing four times with water. For standard adipogenic differentiation, confluent monolayers of MSC were incubated in medium supplemented with 10^−8^ M dexamethasone and 10^−4^ M L-ascorbic acid 2-phosphate (Sigma-Aldrich) with changes of medium every 5 days. After 30 days, the cultures were fixed in 3% formaldehyde in PBS for 10 minutes and stained with Oil Red O.

The phenotype of MSC was analyzed by flow cytometry using a FACScan flowcytometer (BD Biosciences, NJ, USA). The following mAb were used: fluorescein isothiocyanate (FITC)-labeled anti-CD3, anti-B220, anti-CD31, anti-TER119, anti-SCA-1, anti-CD90, phycoerythrin (PE)-labeled anti-CD11b, anti-GR1, anti-CD45, anti-CD11c, anti-Ly6C, and anti-CKIT (BD Biosciences). Isotype controls were used in all cases. To block unspecific antibody binding cells were pre-treated for 30 min at 4°C with saturating concentrations of anti-mouse Fcγ receptor blocking antibodies purified from 24G2 hybridoma supernatants.

### Inflammatory peritoneal macrophages

Thioglycolate-elicited peritoneal macrophages (M) were obtained as previously described [21]. Briefly, 30 g of dehydrated Brewer thioglycolate medium powder (Sigma-Aldrich) was dissolved in 1000 mL deionized water and autoclaved for 20 minutes at 15 pounds of pressure (121°C). The preparation was kept in the dark under sterile conditions at room temperature for at least 6 months before use. Peritoneal exudate cells were elicited by i.p. injection of 2 ml of 3% sterile thioglycolate. Cells, consisting mostly of macrophages (over 90%), were harvested by peritoneal lavage using 5 ml RPMI medium supplemented with 5% FBS, 4 days after intraperitoneal injection of thioglycolate. Cells were plated in 48-well flat-bottom culture plates at 5×10^5^ cells/well in RPMI medium supplemented with 10% fetal bovine serum, 100 IU/ml penicillin and 100 µg/ml streptomycin. After 2 h of incubation at 37°C nonadherent cells were removed by vigorous washing. The purity of macrophage preparations (over 98%) was assessed by flow cytometry using FITC-labeled IgG anti-CD11b (BD Biosciences). The phenotype of macrophages was analyzed using FITC- or PE-labeled mAb directed to CD86, Ia^b^, CD40, H2D^b^, CD36, CD14, and Toll-like receptor 2 (TLR2) (BD Biosciences). Isotype controls were used in all cases. To block unspecific antibody binding, cells were pre-treated for 30 min at 4°C with saturating concentrations of anti-mouse Fcγ receptor blocking antibodies purified from 2.4G2 hybridoma supernatants. When indicated, macrophages were treated with a blocking antibody directed to the IL-10 receptor (10 µg/ml, 1B1.3a, BD Biosciences).

### Co-culture of macrophages and mesenchymal stromal cells

MSC and M were suspended in RPMI medium supplemented with 10% heat-inactivated FCS, 100 IU/ml penicillin and 100 µg/ml streptomycin. MSC were plated in 48-well flat-bottom plates at 5×10^3^ cells/cm^2^. Once cells reached confluence, 5×10^5^ macrophages were added to each well (MSC: M ratio ∼1∶10). Controls include M and MSC cultured alone. Cells were cultured overnight and then incubated for an additional period of 18 h with or without LPS 30 ng/ml. For generation of MSC-conditioned medium, MSC grown to confluence were incubated for 24 h at 37°C. Supernatants were then collected and stored at −20°C until use.

### Analysis of cytokine production by ELISA

M, MSC, and M plus MSC cultured overnight were incubated for an additional period of 18 h with or without LPS, and the presence of the follow cytokines in the supernatants was evaluated by ELISA: TNF-α, IL-6, IL-12p70, IL-23, IL-12p40, IFN-γ (e-Bioscience,CA, USA), and IL-10 (BD Biosciences), following manufacturer's instructions. Production of prostaglandin E2 (PGE2) in the supernatants of MSC was evaluated by ELISA (Cayman Chemical). When indicated, cells were incubated in the presence of acetylsalicylic acid (ASA) or PGE2 (Sigma-Aldrich).

### Analysis of cytokine production by intracellular staining and flow cytometry

To establish the cellular source of the cytokines released in the co-cultures of M and MSC, the production of TNF-α and IL-10 was also analyzed by intracellular staining and flow cytometry. In these experiments, M, MSC, and M plus MSC cultured overnight, were incubated for 18 h with or without LPS. Brefeldin A (10 µg/ml) was added during the last 6 h of culture to inhibit the release of cytokines. Cells were detached with cold-PBS, resuspended in RPMI medium supplemented with 5% FCS, and incubated with saturating concentrations of anti-mouse Fcγ receptor blocking antibodies purified from 2.4G2 hybridome supernatants. Cells were then stained with FITC-labeled antibodies directed to CD11b, washed, fixed, permeabilized and stained with PE-labeled antibodies directed to TNF-α or IL-10 (BD Biosciences).

### Proliferation of OT-II TCD4+ cells induced by OVA-primed macrophages

Thioglycolate-elicited peritoneal macrophages (M) (1×10^5^ cells/100 µl) were cultured overnight in 96 well-flat bottom plates with culture medium alone or MSC-conditioned medium. After washing, cells were treated with LPS (30 ng/ml) in the absence or presence of OVA (500 µg/ml) for 18 hs. After this period, M were washed and fresh culture medium was added. Spleen T CD4+ cells from OT-II mice were purified by negative selection using a T CD4+ cell isolation kit (Miltenyi). Purified T CD4+ cells (>95% purity) were labeled with carboxyfluorescein diacetate succinimidyl ester (CFSE, Molecular Probes) (5 µM, 15 min at 37°C). Cells were washed and analyzed by flow cytometry to be sure that all the cells showed a single fluorescence peak. Macrophages and CFSE-labeled OT-II T CD4+ cells were cultured together (M∶T cell ratio 1∶5) for 72 h. Cells were then harvested and stained with PE-labeled anti-CD4 mAb (BD Pharmingen). Proliferation of the CFSE-labeled T CD4+ cells was analyzed by flow cytometry.

### Quantification of cellular apoptosis and viability by fluorescence microscopy

Quantification was performed by fluorescence microscopy, as previously described [22], using the fluorescent DNA-binding dyes acridine orange (Sigma-Aldrich) (100 µg/ml, to determine the percentage of cells that had undergone apoptosis) and ethidium bromide (Sigma-Aldrich) (100 µg/ml; to differentiate between viable and non-viable cells). With this method, non-apoptotic cell nuclei show ‘structure’, i.e. variations in fluorescence intensity that reflect the distribution of euchromatin and heterochromatin. By contrast, apoptotic nuclei exhibit highly condensed chromatin that is uniformly stained by acridine orange. In fact, the entire apoptotic nucleus are present as bright spherical beads. To assess the percentage of cells showing morphological features of apoptosis, at least 200 cells were scored in each experiment.

### Phagocytosis of apoptotic thymocytes by macrophages

Thymuses were obtained from 6–8-week-old C57BL/6 mice and minced to yield a single-cell suspension. Thymocytes were labeled with carboxyfluorescein diacetate succinimidyl ester (CFSE) (5 µM, 15 min at 37°C). To induce apoptosis, thymocytes were cultured in RPMI medium supplemented with 5% FCS and 2 µM dexamethasone at a concentration of 5×10^6^ cells/ml for 8 h. This treatment yields a population with more than 80% of apoptotic thymocytes and a low degree of contamination by late apoptotic or necrotic cells. M were plated in 48-well flat-bottom culture plates at a concentration of 5×10^5^ macrophages per well, and incubated overnight in RPMI medium supplemented with 10% heat-inactivated FCS, 100 IU/ml penicillin and 100 µg/ml streptomycin, in the absence or presence of MSC (M: MSC ratio 10∶1). Apoptotic tymocytes were then added (M: thymocyte ratio = 1∶10), and cells were incubated for 1 h at 37°C. Cultures were then washed extensively with RPMI and detached with cold PBS. The number of apoptotic thymocytes inside each macrophage was determined by fluorescence microscope using PE-labeled IgG anti-CD11b. At least 200 macrophages were scored in each experiment.

### Phagocytosis of zymosan particles by macrophages

M were plated in 48-well flat-bottom culture plates at a concentration of 5×10^5^ macrophages per well, and incubated overnight in RPMI medium supplemented with 10% heat-inactivated FCS, 100 IU/ml penicillin and 100 µg/ml streptomycin, in the absence or presence of MSC (M: MSC ratio 10∶1). FITC-zymosan (250 µg/ml) was added and phagocytosis of zymosan particles by M was analyzed after 1 h of incubation at 37°C, using PE-labeled IgG anti-CD11b and flow cytometry.

### Infection of macrophages by *Trypanosoma cruzi*

Macrophages (2.5×10^5^/well), were cultured overnight in the absence or presence of MSC (M: MSC ratio = 10∶1) in 8-chamber-slides (Nunc TM, PA, USA). Then, cells were infected with bloodstream trypomastigotes of the *T. cruzi* strain RA [23] at M: *T. cruzi* ratio of 1∶5 for 3 h at 37°C. The cultures were washed five times to remove free parasites and the cells were cultured for an additional period of 45 h at 37°C under 5% CO_2_. Cells were then washed, fixed (4% paraformaldehyde in PBS for 20 min at room temperature) and permeabilized (0.1% Triton X-100 in PBS). Fcγ receptors were blocked using saturating concentrations of anti-mouse Fcγ receptor blocking antibodies purified from 2.4G2 hybridoma supernatants, and the infection of M was determined by analyzing the presence of intracellular amastigotes by immunofluorescence assays using a rabbit polyclonal serum directed to *T. cruzi* and FITC-labeled goat anti-rabbit IgG (Sigma-Aldrich) and PE-labeled antibodies directed to CD11b. Cells were mounted onto microscopic slides using 10% glycerol containing the anti-fade reagent paraphenylenediamine. At least twenty random microscopic fields (400X) and 1000 cells *per* culture were acquired using a Spot RT digital camera attached to a Nikon Eclipse 600 fluorescence microscope (Nikon Inc), supplied with the adequate excitation and emission filters. Cell quantification was performed with the ImageJ open source software developed at the National Institutes of Health (NIH, USA).

### Mice infection by *T. cruzi*

Two-months old C57Bl/6 female mice were infected by intraperitoneal (IP) route with 1×10^5^ bloodstream trypomastigotes of the lethal pantropic/reticulotropic RA strain of *Trypanosoma* cruzi, as previously described [24]. This parasite strain is routinely maintained by weekly passages in CF1 mice. MSC (2.5×10^6^/500 µl pyrogen-free PBS) or PBS (controls) were inoculated by intraperitoneal route at days 4 and 10 post-infection. Parasitemia was measured after the tenth day post-infection. Blood was obtained from a small cut at the end of the tail. The blood was diluted fivefold in red blood cell lysis buffer (150 mM NH_4_Cl, 0.1 mM EDTA, and 10 mM KHCO_3_, pH 7.4), and parasitemia was measured in a Neubauer chamber. Mouse deaths were recorded on a daily basis.

### Inflammatory response induced by s.c. implantation of glass cylinders

A model of chronic inflammation was carried out in C57BL/6 mice, as previously described [25]. Briefly, glass cylinders of 2 cm long, 8 mm wide and around 200 µl internal volume were implanted s.c. into 8-to 12-week-old C57BL/6. It has been shown that the cylinders cause a strong inflammatory process that leads to their infiltration predominantly by macrophages and also to the close of both ends of the cylinders by fibrotic tissue [25]. Two and 7 days after the cylinders were implanted in the mice, 2×10^5^ MSC in 50 µl of pyrogen free-PBS or 50 µl pyrogen-free PBS alone (controls) were inoculated inside the cylinders, using a 22 g needle. After 15 days the liquid content of the cylinders was aspirated using a 22 g needle, centrifuged and the levels of the cytokines TNF-α, IL-12p70, IL-12p40 and IL-10 were determined by ELISA. Cylinders were removed, washed with saline and placed in cold PBS (4°C) for 30 min. Using a specially designed scraper, adherent cells were then removed from the interior of each cylinder (>90% macrophages) to obtain a single cell suspension. The phenotype of adherent macrophages was analyzed by flow cytometry.

### Statistical analysis

Student's paired *t* test was used to determine the significance of differences between mean values, and *p*<0.05 was determined to indicate statistical significance. Differences in the survival of mice infected by T. cruzi were analysed by means of the Kaplan-Meier test followed by log-rank (Mantel-Haenszel) test.

## Results

### MSC characterization

**Figure 1:**
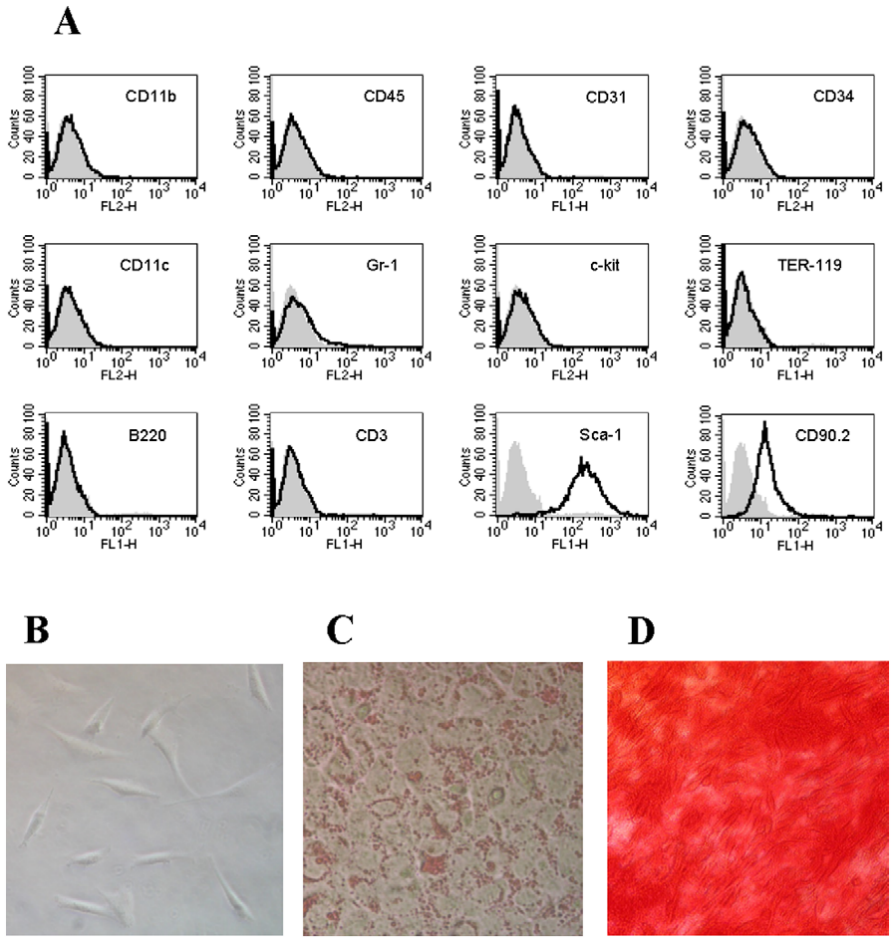
Characterization of bone marrow-derived mesenchymal stromal cells (MSC). MSC were isolated from bone marrow of adult C57BL/6J and cultured using standard protocols. (A) Analysis of the phenotype of MSC by flow cytometry. Grey histograms represent isotype controls (B) Morphology of MSC. (C) Culture of MSC in adipocyte-differentiation media showing cells containing drops of fat revealed by Oil Red O. (D) Culture of MSC in osteogenic-differentiation media showing the formation of calcium containing precipitates stained by Alizarin Red S. A representative experiment is shown.

Mouse MSC were obtained from bone marrow of adult C57BL/6J mice by adherence to plastic culture flasks as previously described [19]. Cells had been passaged from 4 to 7 times were used in all the experiments. Flow cytometric analysis demonstrated that MSC were devoid of typical hematopoietic and endothelial markers. MSC did not express CD11b, CD45, CD31, CD34, CD11c, Gr-1, c-kit, TER-119, B220, CD3, but express MSC-associated antigens such as Sca-1 (stem cell antigen-1) and CD90.2 (**Figure 1A**). Morphologically, these cells had a spindled, fibroblast appearance after expansion (**Figure 1B**). Culture in adipocyte-differentiation media induced the differentiation of MSC into cells containing drops of fat revealed by the colorant Oil Red O, while the culture of MSC in osteogenic-differentiation media results in the formation of calcium containing precipitates which were visualized by staining with Alizarin Red S (**Figures 1C and D**).

### MSC turns inflammatory macrophages into a regulatory profile

**Figure 2:**
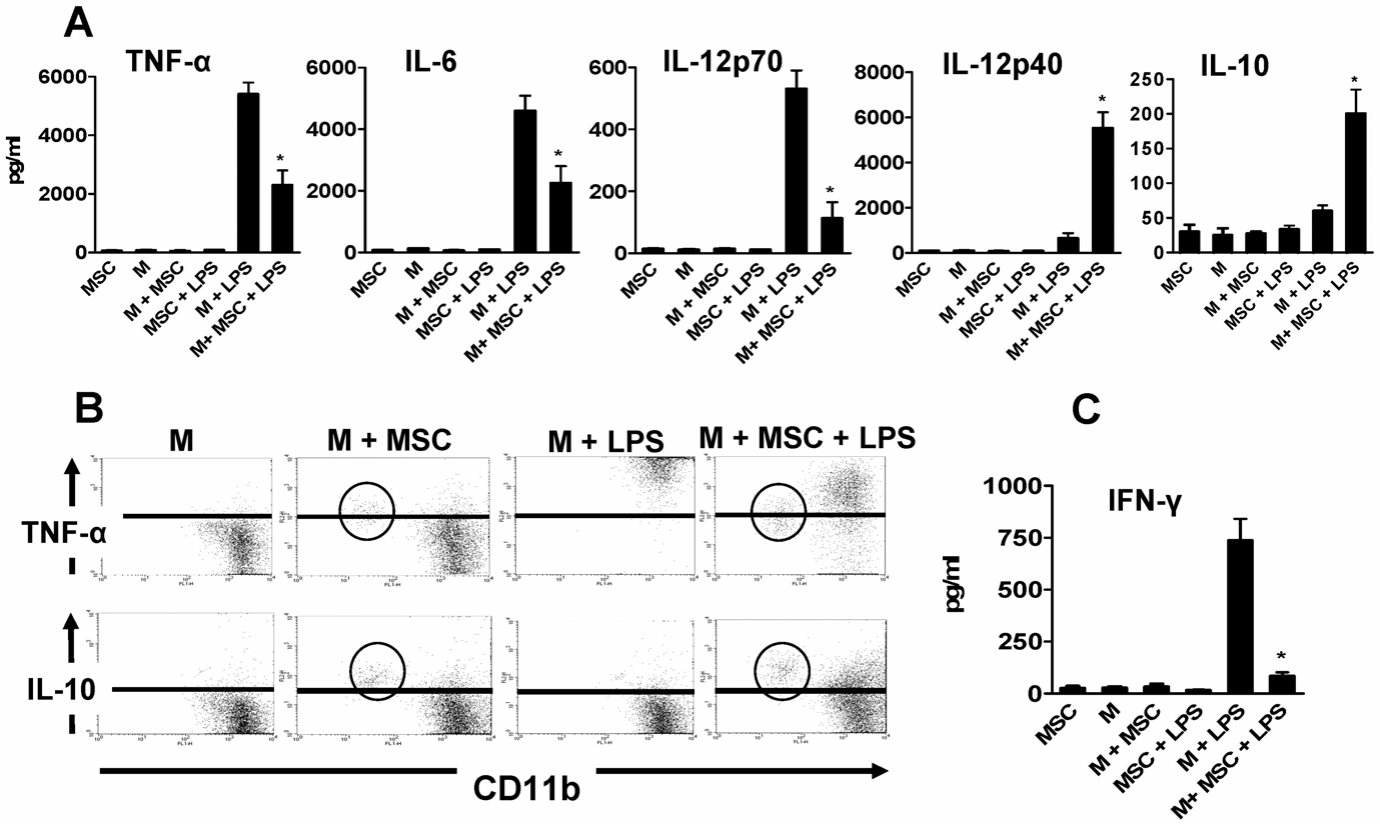
MSC inhibit the production of inflammatory cytokines and enhance the production of IL-10 by thioglycolate-elicited peritoneal macrophages (M). M were cultured overnight in the absence or presence of MSC (M: MSC ratio = 10∶1). Cells were then washed and incubated for 18 h with or without LPS (30 ng/ml), and cytokines were analyzed in cell-supernatants by ELISA (A and C) or by intracellular staining and flow cytometry (B). (A and C) Results are expressed in pg/ml and represent the arithmetic mean ± SEM of 5–6 experiments. (B) Representative dot-plots (n = 4) are shown. Inside the circle MSC. *, p<0.05 for M+MSC+LPS vs M+LPS.

In a first set of experiments we examined the ability of MSC to modulate the production of cytokines by thioglycolate-elicited peritoneal macrophages (M). M were harvested as described under Materials and Methods, and they were cultured overnight in the absence or presence of MSC (M: MSC ratio = 10∶1). Cells were then washed and incubated for an additional period of 18 h with or without LPS (30 ng/ml), and the presence of the cytokines TNF-α, IL-6, IL-12p70, IL-23, IL-12p40 and IL-10 was evaluated in the supernatants by ELISA. The results obtained are shown in **Figure 2A**. Little or no production of cytokines was observed in the cultures of M, MSC or M plus MSC performed in the absence of LPS. Addition of LPS did not stimulate the production of cytokines by MSC but triggered a burst of cytokine production by M. Interestingly, MSC markedly suppressed the production of the proinflammatory cytokines TNF-α, IL-6, and IL-12p70 while increased the production of both IL-12p40 and the anti-inflammatory cytokine IL-10. No production of IL-23 was detected in any condition.

To evaluate the possible contribution of MSC to the production of cytokines observed in co-cultures of M and MSC stimulated by LPS, the presence of TNF-α and IL-10 was analyzed by intracellular staining and flow cytometry. Activation by LPS resulted in the stimulation of TNF-α and IL-10 production by M but not by MSC and, consistent with the analysis of cytokines performed by ELISA in culture supernatants, intracellular staining of M showed that MSC inhibit the production of TNF-α while increased the production of IL-10 (**Figure 2B**).

Interferon-γ (IFN-γ) plays a critical role in innate and adaptive immunity. It is the key cytokine to induce the activation of macrophages by increasing the expression of MHC class II and costimulatory molecules and the production of inflammatory mediators enabling macrophages to display a high anti-microbial and tumoricidal activity [26], [27]. IFN-γ is typically produced by NK cells, NKT cells, TH1 cells and T CD8+ cells [26], [27]. A number of studies have proposed that macrophages are also able to produce IFN-γ [28]-[31]. Our results showed in **Figure 2C** indicated that M produced substantial amounts of IFN-γ upon activation by LPS, and also that the production of IFN-γ was markedly diminished by MSC. While this result reinforces the notion that MSC suppress the production of inflammatory cytokines, the cellular source of IFN-γ in the cultures of M remains uncertain. It should be noted that our preparations of macrophages collected from peritoneal exudates contained between 1.0 and 2.0% of contaminant lymphocytes, and previous studies challenging the assumption that myeloid cells produce IFN-γ demonstrated that the production of IFN-γ by inflammatory peritoneal macrophages could be accounted by the presence of minute numbers of contaminating lymphocytes (<0.4%), some of which express myeloid markers and hence, are not easily distinguishable from macrophages [32], [33].

**Figure 3:**
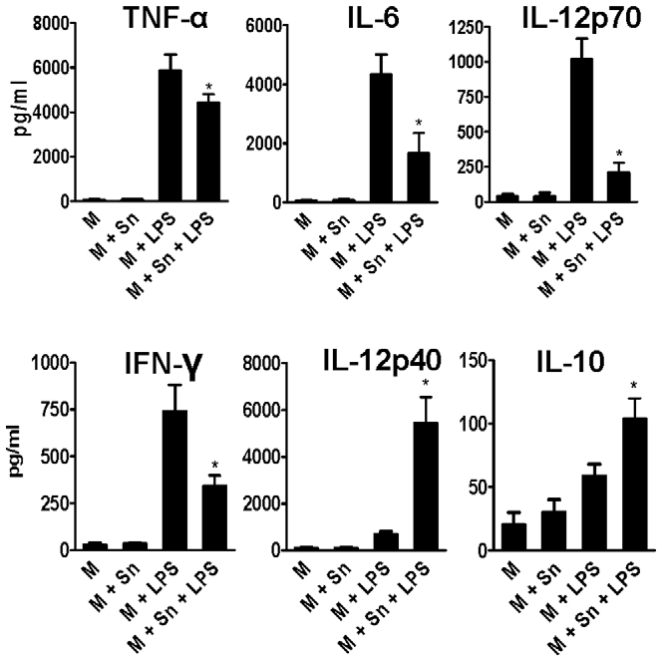
MSC regulate the profile of cytokines produced by activated M through the release of soluble factor(s). MSC grown to confluence were cultured alone for 24 h and the cell-supernatants were then harvested. M were cultured for 18 h with or without LPS (30 ng/ml) in the absence or presence of MSC supernatants (50% V/V) and cytokines were analyzed in cell-supernatants by ELISA. Results are expressed in pg/ml and represent the arithmetic mean ± SEM of 5 experiments. *, p<0.05 for M+Sn+LPS vs M+LPS.

Experiments were then designed to determine whether the modulation exerted by MSC on the production of cytokines by M was dependent on cell-to-cell contact and/or was mediated by soluble factors released by MSC. To this aim, MSC grown to confluence were cultured alone for 24 h and the supernatants were collected. **Figure 3** shows that supernatants from MSC (50% v/v) exerted a modulatory action similar than MSC. In fact, MSC supernatants inhibited the production of the pro-inflammatory cytokines TNF-α, IL-6, IL-12 p70, and IFN-γ while increased the production of IL-12p40 and IL-10 by LPS-activated M. The ability of MSC supernatants to inhibit the production of TNF-α and to stimulate the production of IL-10, however, was lower in comparison with the effect mediated by MSC (see Figure 2A) perhaps reflecting the short half life of the mediator(s) released by MSC and/or the participation of cell-to-cell contact-dependent mechanisms.

**Figure 4:**
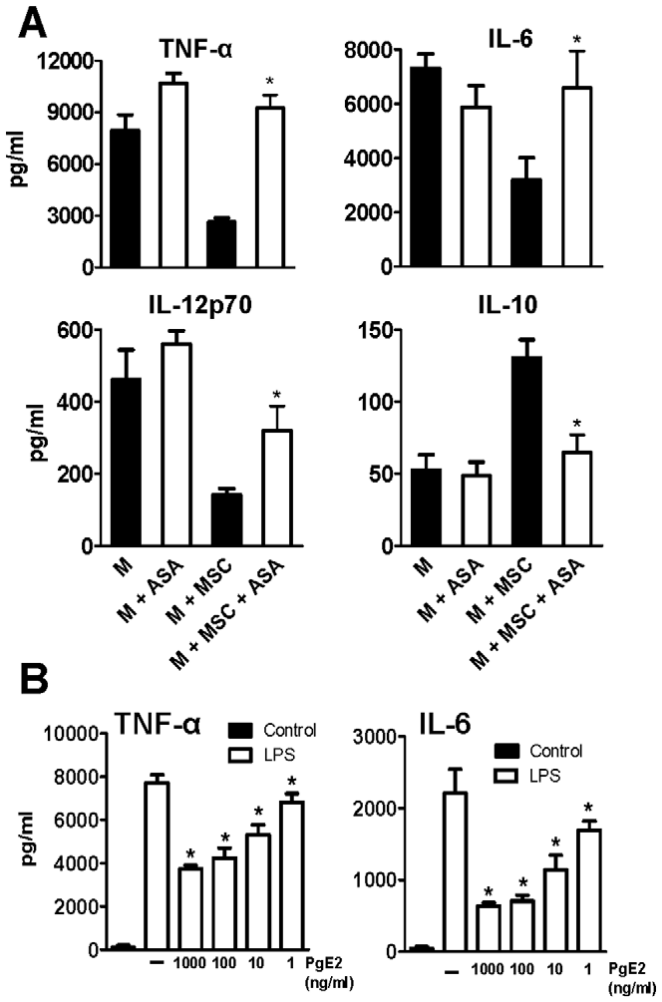
Acetylsalicylic acid (ASA) impairs the ability of MSC to modulate the profile of cytokines produced by M. (A) M were cultured overnight in the absence or presence of MSC (M: MSC ratio = 10∶1) with or without ASA (0.5 mM). Cells were then washed and incubated for 18 h with LPS (30 ng/ml), in the absence or presence of ASA (0.5 mM) and cytokines were analyzed in cell-supernatants by ELISA. (B) M were cultured overnight in the absence (controls) or presence of different concentrations of PGE2. Then, cells were incubated for 18 h with LPS (30 ng/ml) and cytokines were analyzed in cell-supernatants by ELISA. Results are expressed in pg/ml and represent the arithmetic mean ± SEM of 4–5 experiments. *, p<0.05 for M+MSC+ASA vs M+MSC, and for M+LPS+PGE2 vs M+LPS.

Data in **Figure 4** showed that the ability of MSC to down-regulate the production of inflammatory cytokines and up-regulate the production of IL-10 by LPS-stimulated M was strongly inhibited when co-cultures of M and MSC were performed in the presence of acetylsalicylic acid (ASA), a cyclo-oxygenase inhibitor. This suggests that MSC regulate the cytokine profile of activated M through the release of PGE2, one of the major immunomodulator factors produced by MSC [4], [5], [14]. We then analyzed whether MSC constitutively produce PGE2. MSC were grown to confluence and cultured for an additional period of 24 h. Supernatants were collected and the levels of PGE2 were determined by ELISA. MSC supernatants contained 4.3±1.2 ng/ml of PGE2 (mean ± SEM, n = 4). Interestingly, data showed in **Figure 4B** indicated that these concentrations of PGE2 were able to significantly inhibit the production of TNF-α and IL-6 by LPS-stimulated M. On the other hand, since data in Figure 2A showed that MSC constitutively produce low levels of IL-10 we performed another set of experiments using blocking antibodies directed to the IL-10 receptor to determine whether IL-10 secreted by MSC could contribute to the inhibition of the production of inflammatory cytokines by LPS-stimulated M. We observed that treatment of macrophages with anti-IL-10 receptor antibodies (10 µg/ml) did not impair the ability of MSC supernatants to inhibit the production of IL-6 and IL-12p70 by activated macrophages (data not shown).

**Figure 5:**
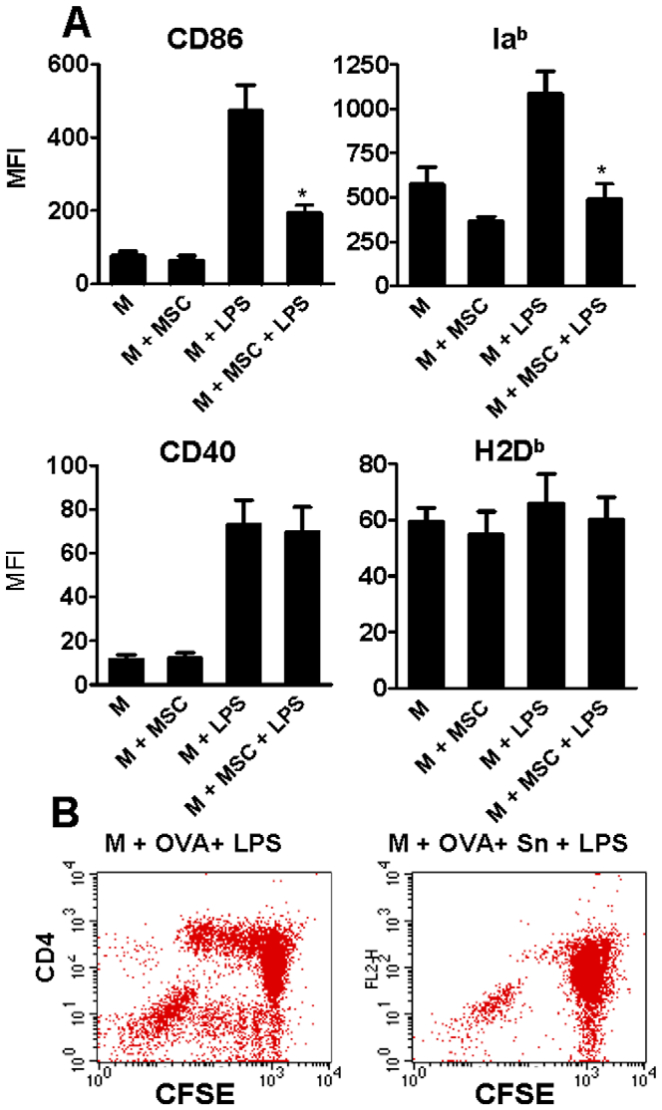
MSC inhibit the up-regulation of CD86 and MHC class II (Ia^b^) in M stimulated by LPS and impairs antigen presentation to T CD4+ cells. (A) M were cultured overnight in the absence or presence of MSC (M: MSC ratio = 10∶1). Cells were then washed and incubated for 18 h with or without LPS (30 ng/ml), and the expression of CD86, MHC class II (Ia^b^), CD40 and MHC class I (H2D^b^) in M was analyzed using FITC-labeled antibodies directed to CD11b and PE-labeled antibodies directed to CD86, Ia^b^, CD40, and H2D^b^, in the gate of CD11b-positive cells. Results are expressed as the mean fluorescence intensity (MFI) values and represent the arithmetic mean ± SEM of 4-5 experiments. *, p<0.05 for M+MSC+LPS vs M+LPS. (B) M (1x10^5^ cells/100 µl) were cultured overnight in 96 well-flat bottom plates with culture medium alone or MSC-conditioned medium (50% V/V). After washing, cells were treated with LPS (30 ng/ml) in the presence of OVA (500 µg/ml) for 18 hs. After this period, M were washed and fresh culture medium was added. Spleen T CD4+ cells from OT-II mice were purified and labeled with CFSE, as described under Materials and Methods. Macrophages and CFSE-labeled OT-II T CD4+ cells were cultured together (M∶T cell ratio 1∶5) for 72 h. Cells were stained with PE-labeled anti-CD4 mAb, and the proliferation of the CFSE-labeled T CD4+ cells was analyzed by flow cytometry. A representative experiment is shown (n = 5). No proliferation was observed when LPS-stimulated M were cultured without OVA.

Not only the production of cytokines but also the phenotype of activated M was regulated by MSC. Results shown in **Figure 5A** indicated that MSC significantly impaired the up-regulation in the expression of both, the costimulatory molecule CD86 and MHC class II (Ia^b^) induced by LPS, without affecting the expression of CD40 and the class I molecule H2D^b^. Similar results were observed with supernatants collected from MSC cultures (data not shown). The ability of MSC to prevent the up-regulation of CD86 and Ia^b^ in LPS-stimulated M supports the notion that MSC might impair the ability of M to act as an antigen-presenting cell. This hypothesis was analyzed using T CD4+ cells isolated from the spleen of OT-II mice, which bear a transgenic αβ T cell receptor specific for the MHC class II-restricted OVA 323–339 peptide. In these experiments, M were cultured overnight in 96 well-flat bottom plates with culture medium alone or MSC-conditioned medium (50% V/V) (Sn). After washing, cells were treated with LPS (30 ng/ml) in the presence of OVA (500 µg/ml) for 18 hs. Then, M were washed and incubated with CFSE-labelled OT-II T CD4^+^ cells for 72 h. Cells were stained with PE-labeled anti-CD4 mAb, and the proliferation of the CFSE-labelled T CD4+ cells was analyzed by flow cytometry. **Figure 5B** shows a representative experiment (n = 5) indicating that supernatants from MSC markedly suppress the ability of LPS-activated M to induce the proliferation of OTII T CD4+ cells (% inhibition = 59±14, n = 5, p<0.05 vs controls).

### MSC stimulate phagocytosis of apoptotic thymocytes by inflammatory macrophages

**Figure 6:**
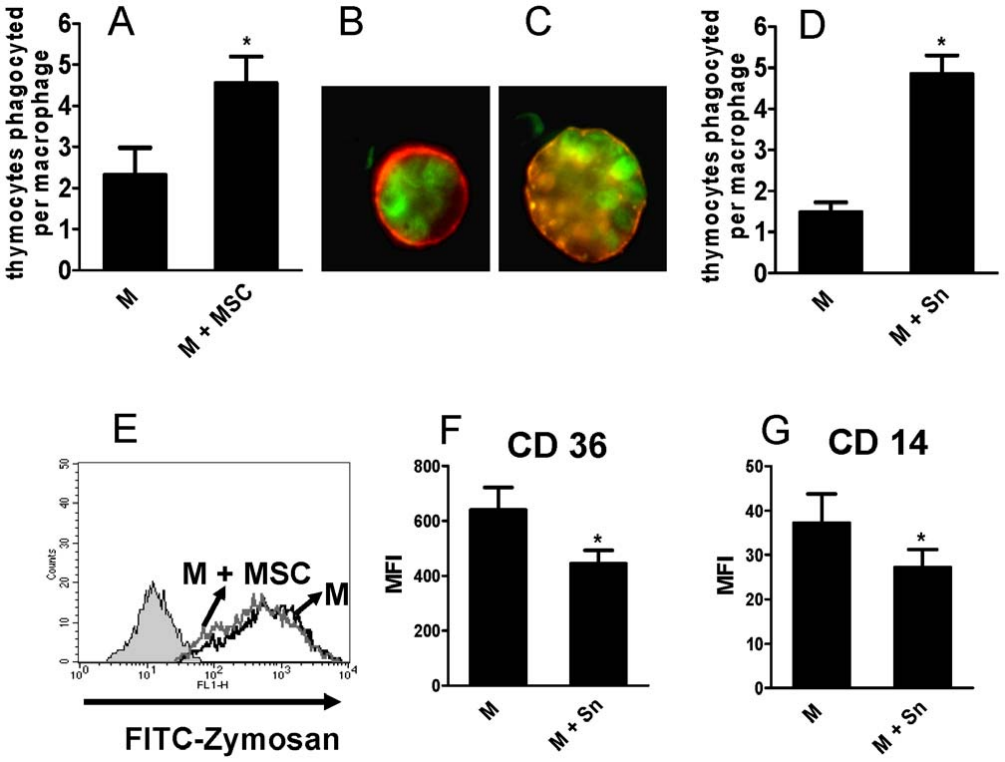
MSC stimulate the uptake of apoptotic thymocytes by M. M were cultured overnight in the absence or presence of MSC (M: MSC ratio 10∶1). Apoptotic thymocytes labeled with CFSE were then added (M: thymocyte ratio = 1∶10), and cells were incubated for 1 h at 37°C. The number of apoptotic thymocytes inside each macrophage was analyzed by fluorescence microscopy using PE-labeled IgG anti-CD11b. At least 200 macrophages were scored in each experiment. (A) Results are expressed as the number of thymocytes internalized per macrophage and represent the arithmetic mean ± SEM of five experiments. *, p<0.05 for M+MSC vs M. (B) Representative images of phagocytosis of apoptotic thymocytes by M cultured alone (B) or in the presence of MSC (C). (D) MSC grown to confluence were cultured alone for 24 h and the cell-supernatants were then harvested. M were cultured overnight with or without MSC supernatants (50% V/V). Apoptotic thymocytes labeled with CFSE were then added (M: thymocyte ratio = 1∶10), and cells were incubated for 1 h at 37°C. The number of apoptotic thymocytes inside each macrophage was analyzed as described above. Results represent the arithmetic mean ± SEM of four experiments. *, p<0.05 for M+Sn vs M. (E) M were cultured overnight in the absence or presence of MSC (M: MSC ratio 10∶1). FITC-labeled zymosan particles (250 µ/ml) were then added and phagocytosis was evaluated after 1 h of incubation at 37°C, using PE-labeled IgG anti-CD11b and flow cytometry, in the gate of CD11b-positive cells. Grey histogram represents the fluorescence of M cultured without zymosan particles. It was similar for M cultured in the absence or presence of MSC. A representative experiment (n = 4) is shown. (F and G) MSC grown to confluence were cultured alone for 24 h and the cell-supernatants were then harvested. M were cultured overnight with or without MSC supernatants (50% V/V). Cells were washed and the expression of CD36 and CD14 was analyzed by flow cytometry. Results are expressed as the mean fluorescence intensity (MFI) values and represent the arithmetic mean ± SEM of 4 experiments. *, p<0.05 for M+Sn vs M.

In the last few years it has become clear that, like dendritic cells and T cells, macrophages display remarkable plasticity. Upon activation they can differentiate into different profiles according the microenvironment stimuli [34]–[37]. The profile of cytokines produced by M cultured with MSC support the notion that M differentiate into a regulatory or alternative profile under the influence of MSC. Since regulatory macrophages might contribute to the resolution of inflammatory processes, not only by releasing anti-inflammatory cytokines but also by promoting the clearance of apoptotic cells [37], [38], we analyzed whether M cultured in the presence of MSC displayed a higher ability to phagocyte apoptotic cells. Experiments were performed by incubating M and MSC (M: MSC ratio = 10∶1) with apoptotic thymocytes (M: thymocyte ratio = 1∶10), for 1 h at 37°C. Phagocytosis was evaluated by fluorescence microscopy using thymocytes labeled with the green fluorescent dye CFSE and macrophages stained with PE-labeled IgG antibodies directed to CD11b. Results in **Figure 6A–C** show that MSC stimulated phagocytosis of apoptotic thymocytes by macrophages. Interestingly, a similar enhancing effect was found using supernatants collected from MSC grown to confluence during 24 h (**Figure 6D**), supporting that stimulation of phagocytosis of apoptotic cells is mediated by the release of factor(s) constitutively produced by MSC. On the other hand, the fact that M cultured with MSC showed no stimulation in their ability to phagocytize zymosan particles (**Figure 6E**) strongly suggests that the increased ability to phagocytize apoptotic cells do not merely reflect a higher endocytic ability of M cultured with MSC. Recognition of apoptotic cells by phagocytes involves a number of receptors able to recognize “eat-me signals” expressed on the surface of apoptotic cells [39]. Among these receptors expressed by macrophages the scavenger receptor CD36 and the glycosylphosphatidylinositol-anchored LPS receptor CD14 appear to play an important role [39], [40]. To analyze whether the ability of MSC to stimulate phagocytosis of apoptotic thymocytes by macrophages was related to an increased expression of receptors able to recognize apoptotic cells we analyzed the expression of CD36 and CD14 in M cultured overnight with MSC supernatants. Results in **Figures 6F and G** show that MSC supernatants did not increase the expression of CD36 and CD14 by macrophages, but rather, they significantly reduced the expression of both receptors.

### MSC markedly increase the susceptibility of macrophages to infection with the parasite Trypanosoma cruzi

**Figure 7:**
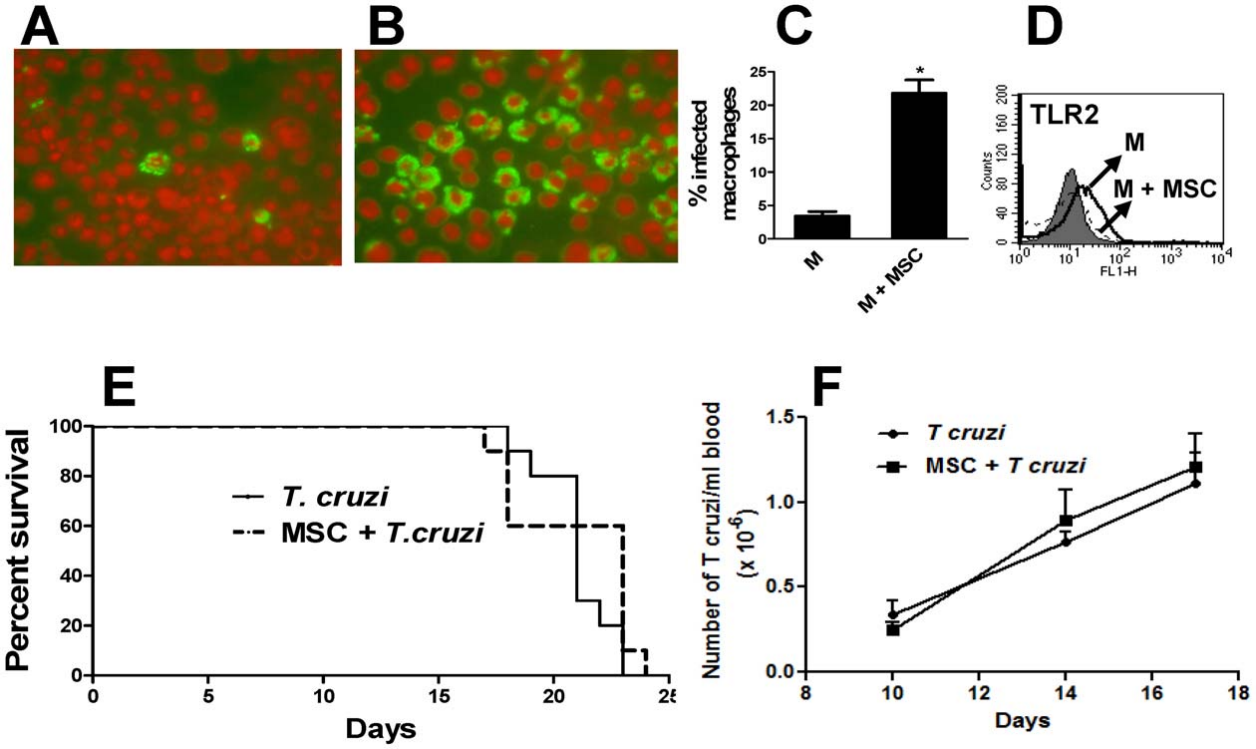
MSC turn M into cells highly susceptible to *T. cruzi* infection. M were cultured overnight in the absence or presence of MSC (M: MSC ratio 10∶1). *T. cruzi* trypomastigotes (M: *T. cruzi* ratio = 1∶5) were then added and incubated for 3 h. Cells were then washed, and cultured for 45 h. Finally, cells were fixed, permeabilized, stained and the presence of intracellular amastigotes was analyzed by fluorescence microscopy as described under Materials and Methods. (A and B) Representative images of intracellular amastigotes in M cultured overnight in the absence (A) or presence (B) of MSC. (C) Results are expressed as the percentage of infected macrophages and represent the arithmetic mean ± SEM of five experiments. *, p<0.05 for M+MSC vs M. (D) M were cultured overnight in the absence or presence of MSC (M: MSC ratio 10∶1). Cells were then washed and the expression of TLR2 in M was analyzed using PE-labeled antibodies directed to TLR2, in the gate of CD11b(FITC)-positive cells. Grey histogram represents the isotype control. A representative experiment (n = 4) is shown. (E and F) Two-months old C57Bl/6 female mice were infected by intraperitoneal (IP) route with 1×10^5^ bloodstream trypomastigotes of the lethal pantropic/reticulotropic RA strain of *T.* cruzi. MSC (2.5×10^6^/500 µl pyrogen-free PBS) or PBS (controls) were inoculated by intraperitoneal route at days 4 and 10 post-infection. (F) Parasitemia was measured at days 8, 14, and 17 post-infection and the results are expressed as the number of *T cruzi*/ml blood (n = 10 for each experimental group). (E) Mouse deaths were recorded on a daily basis. Results are expressed as survival percent (n = 10 for each experimental group).

Infection with the protozoan flagellate parasite *T. cruzi* causes Chagas'disease, a widely distributed infection which represents a major health problem in many Latin American countries. Macrophages are one of the most important targets of *T. cruzi* infection [41]. A large body of evidence indicates that classically activated macrophages play an essential role in host defense against *T. cruzi* by virtue of their ability to destroy intracellular parasites via the production of nitric oxide, a cytotoxic mediator produced by macrophages under the influence of interferon-γ and TNF-α [42], [43]. By contrast, regulatory or alternatively activated macrophages, for example those activated by IL-4, fail to control the infection being super-infected [44]. Because our results support the notion that macrophages differentiate into a regulatory-like profile under the influence of MSC, we examined whether MSC increased the susceptibility of M to *T. cruzi* infection. In these experiments, M cultured overnight alone or in the presence of MSC were infected by exposure to *T. cruzi* trypomastigotes (M: *T. cruzi* ratio = 1∶5) for 3 h. Cells were then washed, and cultured for an additional period of 48 h. Then, cells were fixed, permeabilized, stained and intracellular amastigotes were identified by fluorescence microscopy. Results in **Figure 7A–C** show that the culture of M with MSC resulted in a marked increase (>5-fold) in the percentage of infected M. Supernatants collected from MSC grown to confluence during 24 h failed to reproduce this effect (data not shown). We conclude that MSC turn inflammatory macrophages into cells highly susceptible to *T. cruzi* infection. Previous studies have shown that the resistance of macrophages to the infection by *T. cruzi* is strongly dependent on the expression of toll-like receptors (TLRs) 2 and 9 which recognize *T. cruzi* derived molecules such as GPI anchors and DNA CpG motifs, respectively [45], [46]. To analyze whether MSC might increase the susceptibility of M to *T. cruzi* infection by inhibiting the expression of TLRs, M were cultured overnight in the absence or presence of MSC and the expression of TLR2 was then analyzed by flow cytometry. A representative experiment is shown in **Figure 7D**. A marked reduction in the expression of TLR2 was observed in M cultured with MSC compared with M cultured alone: % inhibition in the expression of TLR2 = 78±16 (n = 4, p<0.05 vs controls). This suggests that the increased susceptibility of M cultured with MSC to the infection by *T. cruzi* may be related to a decreased expression of TLRs.

Additional experiments were performed to analyze whether the ability of MSC to make macrophages more susceptible to *T. cruzi* infection might have an impact in vivo. To this aim, we studied the effect of MSC on the course of *T. cruzi* infection in a murine model. Mice were infected by i.p route with 1×10^5^ bloodstream trypomastigotes of the lethal pantropic/reticulotropic RA strain of *T.* cruzi. Four and eight days post-infection MSC (2.5×10^6^/500 µl pyrogen-free PBS) or PBS (controls) were inoculated by intraperitoneal route. Blood parasitemia was analyzed at days 10, 14, and 17 post-infection, while mouse deaths were recorded on a daily basis. The results obtained are showed in **Figures 7E and F**. They indicated that administration of MSC modified neither parasitemia nor the mortality rate of infected mice.

### MSC promote the recruitment of macrophages and their differentiation into a regulatory-like profile *in vivo*

**Figure 8:**
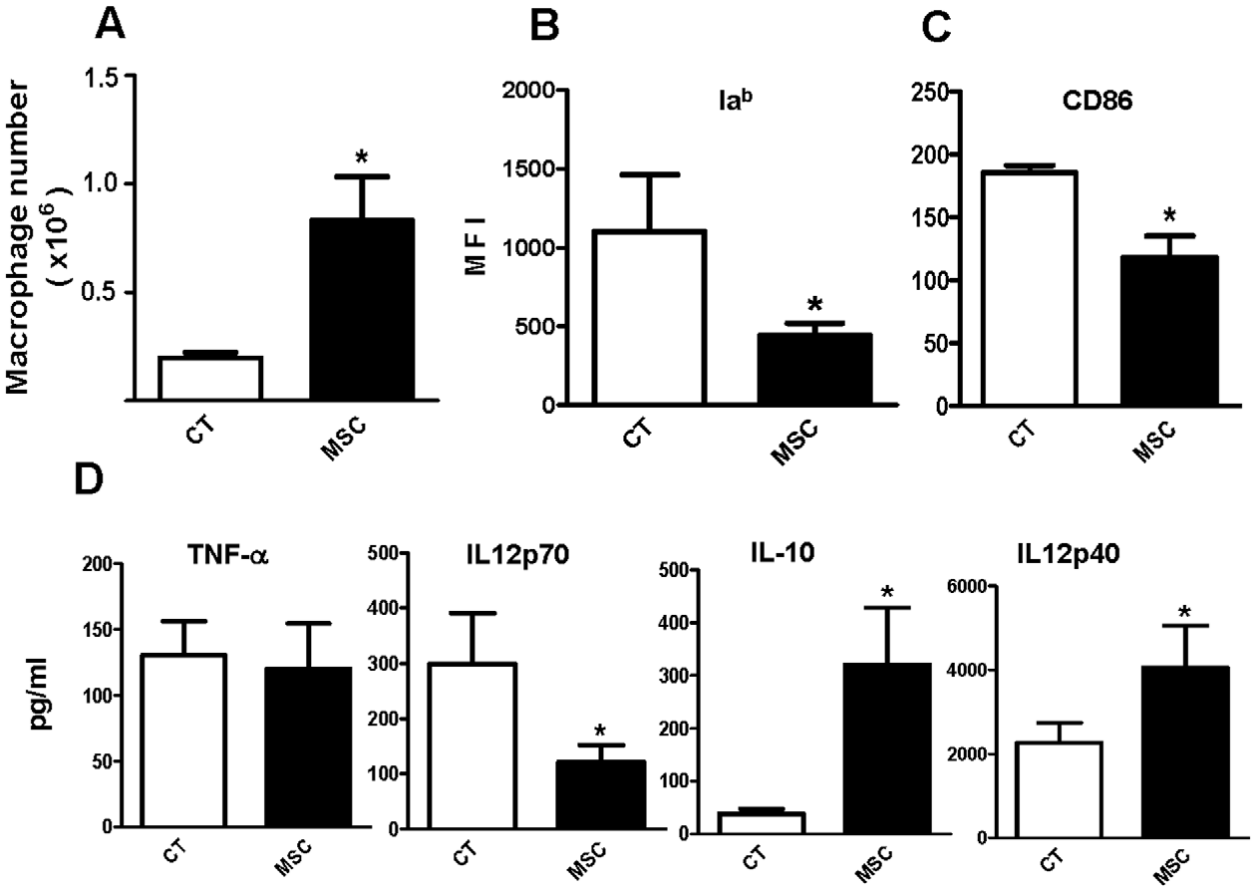
MSC stimulate “in vivo” the recruitment of macrophages and direct their differentiation into a regulatory-like profile. A Glass cylinder of 2 cm long, 8 mm wide and around 200 µl internal volume was implanted s.c. into 8-to 12-week-old C57BL/6 mice. Two and 7 days after the cylinders were implanted in the mice, 2×10^5^ MSC in 50 µl of pyrogen free-PBS or PBS alone (controls) were inoculated inside the cylinders, using a 22 g needle. After 15 days the liquid content of the cylinders was aspirated and the levels of the cytokines TNF-α, IL-12p70, IL-12p40 and IL-10 were determined by ELISA (D). Cylinders were removed, washed with saline and placed in cold PBS (4°C) for 30 min. Adherent cells (>90% macrophages) were removed from the interior of each cylinder to obtain a single cell suspension. The number of the macrophages recruited in each cylinder was measured (A), and their expression of Ia^b^ and CD86 was analyzed by flow cytometry (B-C). Results represent the arithmetic mean ± SEM (n = 12 for each experimental group). *, p<0.05 for Controls (CT) vs MSC.

The lack of effect of MSC on the course of *T. cruzi* infection prompted us to investigate their ability to modulate the profile of macrophages in another experimental model in which MSC were close together with macrophages at the inflammatory foci. To this aim we used a non-infectious model of inflammation induced by s.c. implantation of glass cylinders [25]. These cylinders induce a strong inflammatory response that leads to both, macrophage infiltration and the rapid close of both ends of the cylinders by fibrotic tissue [25, and Isturiz M, personal communication]. One cylinder was implanted in each mice, and 2 and 7 days later 2×10^5^ MSC in 50 µl of PBS or PBS alone (controls) were inoculated inside the cylinders. After 15 days, the liquid content inside the cylinders were harvested and the levels of TNF-α, IL-12p70, IL-12p40 and IL-10 were determined by ELISA. At this time, cylinders were removed from the mice, washed and adherent cells (>90% macrophages) were detached with a cell scraper at 4°C, counted, and characterized by flow cytometry. **Figure 8A** show that MSC enhanced the infiltration of the cylinders by macrophages. In fact, MSC-treated cylinders contained a number of macrophages at least 3-fold higher compared with untreated cylinders. Moreover, these macrophages expressed lower levels of the class II molecule Ia^b^ and CD86 compared with macrophages from untreated cylinders (**Figures 8B and C**). The analysis of cytokines in the liquid harvested from the inside of the cylinders (**Figure 8D**) revealed that MSC-treated cylinders contained lower levels of IL-12 p70 and higher levels of IL-10 and IL-12p40, compared with untreated cylinders. Low levels of TNF-α were detected in both untreated and MSC-treated cylinders.

## Discussion

Mesenchymal stromal cells have emerged as a promising therapeutic tool in regenerative medicine. Although little is known about the in vivo behavior of MSC, they have been introduced in the clinic with encouraging preliminary results [2], [5], [47]. The mechanisms underlying the therapeutic properties of MSC are not well defined. They have been classically related to the stem-cell-like properties of MSC. Recent findings, however, suggest that the therapeutic properties of MSC in a variety of settings are due to their anti-inflammatory and immunomodulatory abilities [2], [4], [5], [47]. In fact, the immunomodulatory effects of MSCs have been validated not only in vitro, but also in vivo, in a number of animal models related to either alloreactive immunity, autoimmunity or anti-tumor immunity [2], [4], [5], [47].

Many studies have been performed to characterize the immunomodulatory effects mediated by MSC. These studies have mainly focused on the action exerted on T and B lymphocytes, dendritic cells, and NK cells [4]–[18]. Little attention has been paid to the analysis of macrophage function. Two recent studies have analyzed this subject. Németh, K et al. [48] showed that administration of MSC to mice before or shortly after inducing sepsis by cecal ligation and puncture reduced mortality and improved organ function. The beneficial effect of MSC was eliminated by macrophage depletion or by blocking antibodies directed to the cytokine IL-10 or the IL-10 receptor. This supports that the therapeutic action mediated by MSC is induced through the stimulation of IL-10 production by macrophages. The authors also showed, in vitro, that MSC increased the production of IL-10 by LPS-stimulated macrophages and demonstrated that this effect is mediated by PGE2 released by MSC. Moreover, Gupta et al. [49] showed that intrapulmonary delivery of bone marrow-derived mesenchymal stromal cells improves survival and attenuates endotoxin-induced acute lung injury in mice. The analysis of cytokines in bronchoalveolar lavage and plasma of endotoxin-challenged mice revealed in MSC-treated mice, lower concentrations of TNF-α and MIP-2 and higher concentrations of IL-10 compared with untreated mice. No differences were found in the levels of PGE2. Because alveolar macrophages are the prominent source of cytokines in this model of acute inflammation the authors also analyzed in vitro whether MSC were able to modulate the production of cytokines by alveolar macrophages stimulated by LPS. They found that MSC exert a low but significant inhibitory effect on the production of TNF-α without affecting the production of MIP-2, while the levels of IL-10 were undetectable. Together, the studies carried out by Németh [48] and Gupta [49] in two different models of acute inflammation support the notion that MSC are able to inhibit the inflammatory response mediated by macrophages through PGE2-dependent or independent mechanisms.

Our observations showing that MSC suppress the production of TNF-α by inflammatory-peritoneal macrophages stimulated by LPS are consistent with the results reported by Németh [48] and Gupta [49], and also by Yang et al. [50]. We also found that other inflammatory cytokines such as IL-6, IL-12p70 and IFN-γ are suppressed under the influence of MSC while the production of IL-10 was significantly enhanced. Of note, the ability of MSC to inhibit the production of the cytokines IL-12p70 and IFN-γ suggest that MSC might be able to suppress inflammation not only by inhibiting the production of pro-inflammatory cytokines during the early step of the inflammatory process, but also by suppressing the induction of Th1 responses which are mainly dependent on IL-12p70 and IFN-γ. We also observed that MSC markedly increase the production of IL-12p40 by LPS-activated M. The p40 chain is the common subunit of IL-12p70 and IL-23, but it also secreted as a p80 homodimer [51]. We did not detect any production of IL-23 by M. This observation together with our results indicating that MSC profoundly inhibits the production of IL12p70 suggests that a large proportion of the p40 produced by LPS-activated M under the influence of MSC might be secreted as a p80 homodimer. Interestingly, it has been suggested that the p80 homodimer binds to the IL-12 receptor and acts as a potent IL-12p70 antagonist [52], [53].

Our results suggest that MSC switch the macrophages to an anti-inflammatory profile not only by suppressing the production of inflammatory cytokines enhancing the production of IL-10, but also by stimulating the phagocytosis of apoptotic cells, a novel mechanism through which MSC might contribute to ameliorate tissue injury. Interestingly, all these effects were induced by either MSC or supernatants collected from unstimulated MSC cultured alone, indicating that the modulatory action exerted by MSC on macrophage function is largely mediated by factors constitutively produced by MSC. This contrasted with previous studies directed to characterize certain immunomodulatory effects mediated by MSC on T and B lymphocytes, since they appeared to require some degree of MSC activation [4], [5], [14].

The clearance of apoptotic cells plays a critical role in the resolution of inflammatory processes [54]. In fact, inflammation usually involves the local recruitment of large number of leukocytes which undergo apoptosis at the inflammatory foci. If apoptotic cells are not efficiently removed they release intracellular constituents able to further increase the course of inflammation, among them, ATP, K^+^ ions, uric acid, high-mobility group box 1 protein (HMGB1) and several members of the S100 calcium-binding protein family [55]. The mechanisms through which MSC increase phagocytosis of apoptotic thymocytes by macrophages remain to be determined. MSC were completely unable to increase the uptake of zymosan particles by macrophages, supporting that the increased ability of macrophages to phagocytize apoptotic cells is not due to an improved efficiency of their phagocytic machinery. Moreover, two important receptors involved in the recognition of apoptotic cells, CD36 and CD14, were down-regulated in M cultured under the influence of MSC. It should be noted, however, that the uptake of apoptotic cells by professional phagocytes depends, besides CD36 and CD14, on a complex system of receptors [39], [40], some of which might be up-regulated in macrophages under the influence of MSC.

Of note, we found that MSC induce a marked increase in the susceptibility of macrophages to infection with the protozoan flagellate parasite *T. cruzi* the etiologic agent of Chagas'disease [41]. The mechanisms responsible for the inherent relative resistance of macrophages to infection by *T. cruzi* appear to be linked to the production of the inflammatory cytokines TNF-α, IL-12p70, and IFN-γ which drive nitric oxide production [41]–[44]. Our results showing that MSC down-regulated the production of all of these cytokines in LPS-stimulated macrophages suggest that a diminished ability to produce inflammatory cytokines might be the reason for the increased susceptibility to infection. Interestingly, and contrasting with the suppression of inflammatory cytokines found in LPS-stimulated macrophages, we observed that supernatants from MSC failed to increase the susceptibility of macrophages to *T. cruzi*-infection (unpublished results) suggesting that cell-to-cell contact-dependent mechanisms are involved. Previous studies have shown that when macrophages and *T. cruzi* are co-incubated in vitro, macrophage resistance to infection requires the expression of TLRs 2 and 9 which recognize *T. cruzi*-derived pathogen-associated molecular patterns (PAMPs) leading to the activation of potent microbicidal mechanisms [45], [46]. We did not study the expression of TLR9 but found that MSC induced a marked decrease in the expression of TLR2 which might explain, at least in part, the enhanced macrophage susceptibility to *T. cruzi* infection. On the other hand, in the experiments directed to evaluate whether the adoptive transfer of MSC to *T. cruzi* infected mice resulted in a more aggressive course of infection, we failed to detect any effect. Not only parasitemia levels but also the rates of death of infected mice were similar in untreated and MSC-treated mice. Since MSC appear to enhance the susceptibility of macrophages to *T. cruzi* infection by cell-to cell contact-dependent mechanisms, this results might reflect the inability of MSC to reach the major sites of parasite multiplication such as the spleen and the liver.

Guided by this presumption, we then analyzed in vivo the immunomodulatory activity of MSC in another experimental model in which MSC were close together with macrophages during the course of an inflammatory response. Subcutaneous implantation of glass cylinders induces a strong inflammatory response leading to both, macrophage infiltration in the interior of the cylinders and the rapid occlusion of both ends of the cylinders by fibrotic tissue [25], thus favoring the retention of transferred MSC inside the cylinders. In this model, we found that MSC enhanced the infiltration of the glass cylinders by macrophages. Consistent with our in vitro observations, these macrophages showed low levels of the class II molecule Ia^b^ and CD86. Moreover, the analysis of the liquid content of the cylinders revealed that MSC-treated cylinders contained lower levels of IL-12 p70 and higher levels of IL-10 and IL-12p40, compared with untreated cylinders. Low levels of TNF-α were found in both untreated and MSC-treated cylinders. Together, these results support the notion that during the course of an inflammatory response MSC might favor the resolution of inflammation via the recruitment and differentiation of macrophages into a regulatory-like profile.

Our results show that MSC impose dramatic changes in the function of macrophages inhibiting the production of anti-inflammtory cytokines, promoting their ability to phagocytize apoptotic cells and enhancing their susceptibility to *T. cruzi* infection. Interestingly, each of the changes appears to involve, at least partially, different mechanisms. Experiments performed with ASA and PGE2 support a relevant role for PGE2 in the ability of MSC to modulate the cytokine profile of activated macrophages. By contrast, PGE2 does not appear to be involved in the stimulation of the phagocytosis of apoptotic cells by macrophages. In fact, previous studies have already shown that PGE2 did not modify [56] or significantly suppress [57], the uptake of apoptotic cells by macrophages. Consistent with these observations, we found that PGE2 did not increase the uptake of apoptotic thymocytes by macrophages (Maggini J, unpublished observation). Finally, the enhanced susceptibility of macrophages to *T cruzi* infection induced by MSC appeared to be mainly dependent on mechanisms that require the physical interaction between MSC and macrophages. Thus, our results suggest that MSC display different mechanisms to modulate the functional profile of macrophages.

Not only dendritic cells and T cells, but also macrophages, display a remarkable plasticity and can dramatically change their physiology in response to environmental stimuli. Mosser and Edwards [37] have recently proposed three major populations of macrophages based on three different homeostatic activities; host defense (classically-activated macrophages), wound healing (wound-healing macrophages) and immune regulation (regulatory macrophages). Our results support the notion that under the influence of MSC, classically activated macrophages turn into wound-healing or regulatory macrophages, suggesting a novel mechanism through which MSC might modulate the course of the immune response.
